# Expanding the clinical spectrum of *MTTF* mutations

**DOI:** 10.1016/j.ymgmr.2019.100501

**Published:** 2019-08-13

**Authors:** Giulia Barcia, Zahra Assouline, Alessandra Pennisi, Julie Steffann, Nathalie Boddaert, Cyril Gitiaux, Agnès Rötig, Jean-Paul Bonnefont, Arnold Munnich

**Affiliations:** Fédération de Génétique et Institut Imagine, Université Paris Descartes, Hôpital Necker Enfants Malades, Paris

## Abstract

We report on a *de novo* m.586G > A *MTTF* mutation in a 14 yrs old boy with non-progressive muscle weakness, myalgia, normal brain MRI, normal schooling and absent central nervous system involvement. The same m.586G > A *MTTF* mutation has been previously reported in a 57 yrs-old woman with a progressive neurodegenerative disorder, akinesia-rigidity, abnormal movements, dementia, and psychiatric disorder. Those two strikingly different clinical presentations emphasize the impact of either mitochondrial factors (heteroplasmy, mitotic segregation) or hitherto unknown nuclear factors on the clinical expression of genetically homogeneous mtDNA mutations.

Mitochondrially encoded tRNA phenylalanine (tRNA^Phe^*, MTTF*) is a transfer RNA that transfers phenylalanine residues to growing polypeptide chains at the ribosome site of protein synthesis during mitochondrial translation. Mutations in *MTTF* cause multiple mitochondrial respiratory chain deficiency and have been associated to a variety of clinical presentations, including Myoclonic Epilepsy with Ragged-Red fibers (MERRF) [1], rhabdomyolysis [2], Mitochondrial Encephalomyopathy, Lactic Acidosis with Stroke-like episodes (MELAS) [3], juvenile myopathy, encephalopathy, lactic acidosis and stroke [4], and recently to a neurodegenerative disorder with psychiatric disturbance, dementia and akinesia-rigidity (m.586G > A *MTTF* mutation) [5].

We have observed the same m.586G > A *MTTF* mutation in a 14 yrs. old boy presenting with non-progressive muscle weakness and myalgia, normal schooling and neither brain MRI anomalies nor central nervous system involvement.

The patient was the unique child of healthy unrelated parents with an unremarkable family history. He was born after a term pregnancy and normal delivery (birthweight: 2535 g; height: 49.5 cm; OFC: 34 cm). His developmental milestones were normal, he could walk and start speaking aged 14 mths and did well at school in his first years of life. At 8 yrs., he started complaining about muscle pain, night cramps, fatigue after short walks (<5 min). He also complained about abdominal pain, headache and vomiting following physical exertion. He had a reduced muscle bulk, reduced lower limb strength (2/5), trunk and girdle weakness and difficulties to sit unaided. He had brisk deep tendon reflexes but normal eye movements, absent cranial nerve involvement and no lid ptosis. At 14 yrs., he is a normally intelligent, dedicated college student.

Brain MRI and heart ultrasound were normal. Metabolic work up consistently showed elevated plasma lactate and lactate/pyruvate molar ratios (lactate: 4, 8–6, 9 mM, L/P ratio: 61), normal plasma creatine kinase and amino acids and trace amounts of urinary Krebs cycle intermediates (3-methylglutaconic acid, 3-hydroxyglutaric acid). Electrophysiology of the deltoid detected overt muscle involvement with no signs of peripheral neuropathy (normal motor/sensory nerve conduction velocity). Histological and histochemical analyses of his muscle revealed a typical aspect of mitochondrial myopathy with numerous ragged red fibers on modified Gomori trichrome, COX-negative, SDH-positive fibers, with lipid and glycogen storage and no evidence of myocyte necrosis or regeneration.

Spectrophotometric analyses of mitochondrial enzyme activities on skeletal muscle homogenate showed elevated citrate synthase (CS) and respiratory chain complex II and V activities (CII, CV), suggestive of an increased mitochondrial mass. Complexes I, III and IV were severely defective (CI/CS: 0.03, normal: 0.12–0.22; CIII/CS: 0.3, normal: 2.3–3.6; CIV/CS: 0.1, normal: 1.2–2). High throughput sequencing of long-range PCR-amplified mitochondrial DNA (mtDNA) detected a heteroplasmic m.586G > A variation in *MTTF* with high levels of mutant species in skeletal muscle (>90%) and urinary epithelia (40%) while barely detectable mutations were found in circulating leukocytes (4%). No other variation was detected on sequencing the entire mitochondrial genome. No detectable levels of the m.586G > A *MTTF* variation were found in circulating leukocytes and urinary epithelia of his mother. This variation affects a nucleotide that is phylogenetically conserved in the tRNA^Phe^ sequence (http://mamit-trna.u-strasbg.fr), located in the D-stem and never reported as a polymorphism (Mitomap database) [6].

A variation located 3 nucleotides upstream in the MT-TF gene (m.583G > A) has been reported in a case of acute episodes of headaches, photophobia and vomiting similar to that of our patient, and a fully recovered left arm focal motor fitting [7]. Another variation located 4 nucleotides upstream in the MT-TF gene (m.582 T > C) is reportedly associated with mitochondrial myopathy [8]. Most interestingly, a mtDNA variation identical to the one found in our patient (m.586G > A *MTTF* mutation) has been reported in 57 yrs-old woman with a progressive neurodegenerative disorder characterized by akinesia-rigidity, abnormal movements, dementia, and psychiatric disorder [5]. She developed reduced mobility and an increasing number of falls over a period of 8 years. During the previous year, she had been able to walk only short distances. Additionally, her short-term memory and word-finding ability had deteriorated markedly and she had paranoid delusions and auditory hallucinations. Her muscle biopsy revealed ragged-red fibers and numerous COX-deficient fibers (30%) but no enzymatic analyses were possible [5].

The difference in presentation, age of onset and clinical course of the two patients carrying an identical m.586G > A *MTTF* mutation is striking. It is conceivable that either intrinsic mitochondrial genetic factors (such as heteroplasmy and mitotic segregation) or hitherto unknown nuclear factors account for those discrepancies.

In conclusion, this observation expands the range of clinical phenotypes associated with *MTTF* mutations and contributes to a growing list of clinical phenotypes associated to mutations in this mitochondrial tRNA gene. Moreover, it emphasizes the impact of heteroplasmy, mitotic segregation or hitherto unknown nuclear genes on the clinical expression of genetically homogeneous mtDNA mutations.
